# Supplementary material for: Two Decades of Brain Tumour Imaging with O-(2-[18F]fluoroethyl)-L-tyrosine PET: The Forschungszentrum Jülich Experience
Source: Cancers (Basel). 2022 Jul 8;14(14):3336. doi: 10.3390/cancers14143336 (PMC9319157; doi:10.3390/cancers14143336)
Supplement: Supplementary file 1 [file cancers-14-03336-s001.zip › cancers-1773575-supplementary.pdf]

Evaluation of ^{18}F-Fluorethyltyrosine PET (FET-PET) in Brain Tumor Diagnosis

Dear colleague,

The purpose of this study is to statistically analyse the use of FET-PET in brain tumour diagnostics. An important aspect is the perspective of the referring neurosurgeons, neuro-oncologists and radiotherapists. We would be very grateful if you could answer the following questions. The questions assume that conventional MR diagnostics (T1, T1+KM, FLAIR) are already available. If a question is not relevant to your field, please leave the field open.

How do you assess the additional value of FET PET in comparison to conventional MRI in the:

Initial diagnosis of cerebral gliomas or unclear brain lesions

Very important	1	2	3	4	5	Unimportant
1. Confirmation of suspected glioma	○	○	○	○	○	
2. Estimation of tumor extent	○	○	○	○	○	
3. Biopsy guidance	○	○	○	○	○	
4. Plannin of surgery and radiotherapy	○	○	○	○	○	

Early postoperative diagnosis of cerebral gliomas (1 - 2 months post-op)

Very important	1	2	3	4	5	Unimportant
5. Tumor residuals (yes/no)	○	○	○	○	○	
6. Estimation of tumor extent	○	○	○	○	○	

Recurrence diagnostics of cerebral gliomas

Very important	1	2	3	4	5	Unimportant
7. Suspected recurrence (yes/no)	○	○	○	○	○	
8. Detection of pseudoprogression	○	○	○	○	○	
9. Extent of the recurrent tumor	○	○	○	○	○	
10. Planning of surgery/radiotherapy	○	○	○	○	○	

Therapy monitoring of cerebral gliomas

Very important	1	2	3	4	5	unimportant
11. Temozolomide	○	○	○	○	○	
12. PCV scheme	○	○	○	○	○	
13. Antiangiogenic therapy	○	○	○	○	○	
14. Immunotherapy	○	○	○	○	○	
15. Other therapies	○	○	○	○	○	

Diagnostics of brain metastases

	Very important	1	2	3	4	5	unimportant
16.	Diagnosis of recurrence	○	○	○	○	○	
17.	Therapy monitoring	○	○	○	○	○	

In what percentage of your patients (approx.) do you consider FET-PET useful or necessary for the various indications?

		<10%	25%	50 %	75%	>90 %
18.	Differential diagnosis of brain lesion	○	○	○	○	○
19.	Prognosis of gliomas	○	○	○	○	○
20.	Biopsy guidance in gliomas	○	○	○	○	○
21.	Tumor extent for OP/RT planning	○	○	○	○	○
22.	Diagnosis of recurrent gliomas	○	○	○	○	○
23.	Therapy monitoring in gliomas	○	○	○	○	○
24.	Diagnosis of recurrent brain metastasis	○	○	○	○	○
25.	Therapy monitoring of brain metastasis	○	○	○	○	○

How do you assess the value of advanced MR procedures (PWI, MRS, DWI) for the indications mentioned compared to FET PET?

	Very important	1	2	3	4	5	unimportant
26.	Differential diagnosis of brain lesion	○	○	○	○	○	
27.	Diagnosis of recurrent gliomas	○	○	○	○	○	
28.	Therapy monitoring in gliomas	○	○	○	○	○	
29.	Diagnosis of recurrent brain metastasis	○	○	○	○	○	
30.	Therapy monitoring of brain metastasis	○	○	○	○	○	

How do you rate the following statements:

	correct	1	2	3	4	5	not correct
31. The availability of FET PET is very important for me		○	○	○	○	○	
32. The availability of FET PET is sufficient for my needs		○	○	○	○	○	
33. FET PET should be approved as a standard procedure		○	○	○	○	○	
34. FET PET should be available in specialized neuroongological centers only		○	○	○	○	○	

Further comments:
